# Computer-Aided Drug Design towards New Psychotropic and Neurological Drugs

**DOI:** 10.3390/molecules28031324

**Published:** 2023-01-30

**Authors:** Georgia Dorahy, Jake Zheng Chen, Thomas Balle

**Affiliations:** 1 Sydney Pharmacy School, Faculty of Medicine and Health, The University of Sydney, Sydney, NSW 2006, Australia; 2 Brain and Mind Centre, The University of Sydney, Camperdown, NSW 2050, Australia

**Keywords:** structure-based drug design, ligand-based drug design, artificial intelligence, docking, QSAR, pharmacophore, deep learning, molecular dynamics, Alzheimer’s disease, schizophrenia, neuropathic pain, neurological, psychotropic, virtual screening, computer-aided drug design

## Abstract

Central nervous system (CNS) disorders are a therapeutic area in drug discovery where demand for new treatments greatly exceeds approved treatment options. This is complicated by the high failure rate in late-stage clinical trials, resulting in exorbitant costs associated with bringing new CNS drugs to market. Computer-aided drug design (CADD) techniques minimise the time and cost burdens associated with drug research and development by ensuring an advantageous starting point for pre-clinical and clinical assessments. The key elements of CADD are divided into ligand-based and structure-based methods. Ligand-based methods encompass techniques including pharmacophore modelling and quantitative structure activity relationships (QSARs), which use the relationship between biological activity and chemical structure to ascertain suitable lead molecules. In contrast, structure-based methods use information about the binding site architecture from an established protein structure to select suitable molecules for further investigation. In recent years, deep learning techniques have been applied in drug design and present an exciting addition to CADD workflows. Despite the difficulties associated with CNS drug discovery, advances towards new pharmaceutical treatments continue to be made, and CADD has supported these findings. This review explores various CADD techniques and discusses applications in CNS drug discovery from 2018 to November 2022.

## 1. Introduction

Mental disorders including neurological and psychiatric disorders represent an area of medicine where there is a considerable unmet need for new and more advanced treatments. Diseases such as Alzheimer’s disease and Parkinson’s disease only have treatments available that provide symptomatic relief [1,2]. As these diseases progress, the efficacy of current therapies wanes and is no longer able to manage these conditions. Mental illnesses such as schizophrenia have several treatment options available; however, they are associated with a plethora of adverse drug reactions that can severely affect a patient’s physical health due to cardiometabolic syndrome [3]. For other neurological conditions, such as brain injuries, there is no treatment or cure. Thus, designing drugs to prevent or halt neuronal death and subsequent deficits is necessary and urgent. 

The discovery of new drugs targeting mental disorders is associated with some of the highest fail-rates in drug discovery, with 85% of drugs failing in phase II and III clinical trials [4]. This makes the development of central nervous system (CNS) drugs extremely expensive, given their tendency to fail in later stage trials [5], with an estimated cost of upwards of $2 billion to bring a drug to market in 2019 [6]. Ensuring the best possible starting point for new discovery projects is imperative, and computer-aided drug design (CADD) techniques are important in this context. These methods are an attractive starting point for new projects and have become one of the mainstays in the early drug discovery process given their reduced time and labour intensity in comparison to traditional drug design and laboratory testing. Thus, CADD can help to shorten the time from initial research to bringing a drug to market and alleviate the high associated costs. 

**Figure 1:**
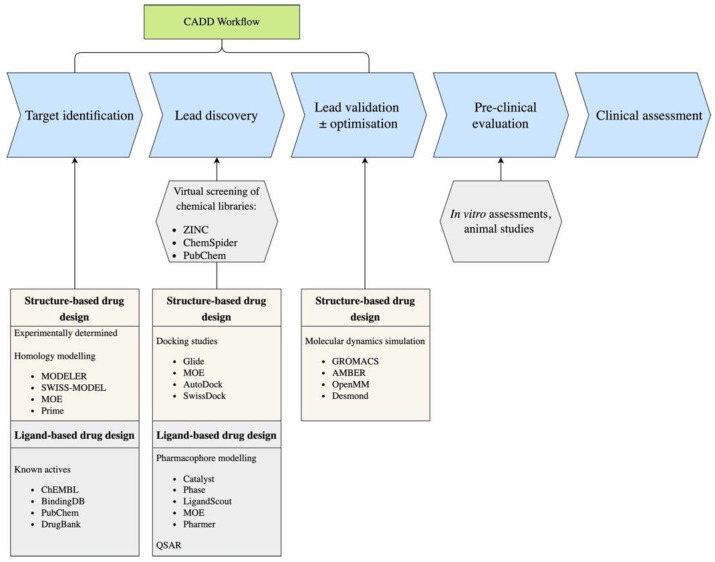
A drug design workflow including the stages of CADD. Both structure- and ligand-based applications are outlined. A sample of chemical libraries and software applications used in the different stages of the workflow are highlighted. It must be noted that these lists are not exhaustive and other libraries and software applications are available for use.

CADD is typically classified into ligand-based and structure-based methods (Figure 1). Ligand-based methods work on the principle that the chemical structure of a drug is related to its biological activity. Thus, with a series of known active and inactive ligands at hand, structure activity relationships (SARs) can be derived and used to predict new and better molecules. The main challenge in ligand-based drug design is how to describe chemical structure. This can be done to various levels of sophistication ranging from 2D to 3D descriptors. Structure-based drug design methods rely on the knowledge of the 3D structure of the biological target. This provides an insight into the binding site architecture which may then be utilised to assess if a ligand would make a suitable lead molecule based on the binding site interactions. Both ligand- and structure-based methods rely on goodness of fit for small molecules to select compounds that may be best suited for further research. Molecules are scored according to features such as similarity, correlations to specific molecular properties, or binding energy, making them ideal techniques for the early stages of the drug discovery process. Although these techniques will be discussed separately, it is important to note that they are often used in concert to yield more accurate results and to reduce the computational burden when chemical libraries being screened are large. The result is a complimentary process towards new chemical entities.

In this review, we summarise the most common CADD methods, including homology modelling, molecular docking, molecular dynamics simulations, pharmacophore modelling, quantitative-structure-activity relationship (QSAR) methods, and the more recent deep learning (DL) applications that have proven their efficacy in CADD. Furthermore, we provide examples of their application in drug discovery projects for psychiatric and neurological conditions. The selected examples are from 2018 to January 2023 and focus on applications that are supported by experimental data to demonstrate the validity and value of the in-silico methods applied. The examples cover a wide range of biological targets including cannabinoid receptor 1, acetylcholinesterase and the α7 nicotinic acetylcholine receptor. We demonstrate that CADD is an important tool in the discovery of CNS drugs, especially during the early stages of the drug discovery pipeline. 

## 2. Ligand Based Techniques

Ligand-based drug design (LBDD) uses data from a known ligand or set of ligands to anticipate the activity of other small molecules based on the commonality with the molecules being investigated. This works on the principle that compounds with a similar chemical structure will exhibit similar binding properties.

### 2.1. 2D Based Methods

In 2D based methods, the chemical structure is represented as a set of descriptors, which are then used to screen for molecules possessing similar properties. A range of physicochemical properties and molecular features are calculated from a 2D molecular graph [7]. These properties, including molecular weight and logP [8], are essential in assessing the suitability of a molecule as a drug based on Lipinski’s rule of five [9]. By extension, the physicochemical properties provide information about how likely a ligand is to transverse the blood–brain barrier (BBB) [10], which is an integral consideration in CNS drug development. Molecular features, such as topological indices, fragment counts and substructure counts can also be ascertained from a 2D molecular graph. The molecular and physicochemical properties provide the data needed to find new lead compounds using techniques such as similarity searching [11,12]. A set of molecular descriptors will be defined to screen for the ligands which best match and thus make suitable candidates for further research. Although details of the 3D molecule are not considered with this technique, it is an attractive approach for ultra-large chemical libraries due to the reduced computational burden associated with screening for hit compounds.

### 2.2. Pharmacophore Modelling

A pharmacophore model is a collection of chemical features that defines the interactions of a ligand with a biological target that elucidates a physiological response [13]. Pharmacophore modelling can also be used to differentiate whether a ligand would behave as an agonist or antagonist. This technique is particularly useful where detailed information of the structure of the target is not available. Furthermore, indirect information about binding site features based on the molecular properties of known active molecules can be inferred from pharmacophore modelling. Pharmacophore modelling was applied in search for antagonists of the α7 nicotinic acetylcholine receptor (nAChR) prior to the 3D structure of this protein being solved. A high potency lead, T761-0184, was uncovered, providing a novel lead for the treatment of CNS disorders such as schizophrenia [14]. 

**Figure 2:**
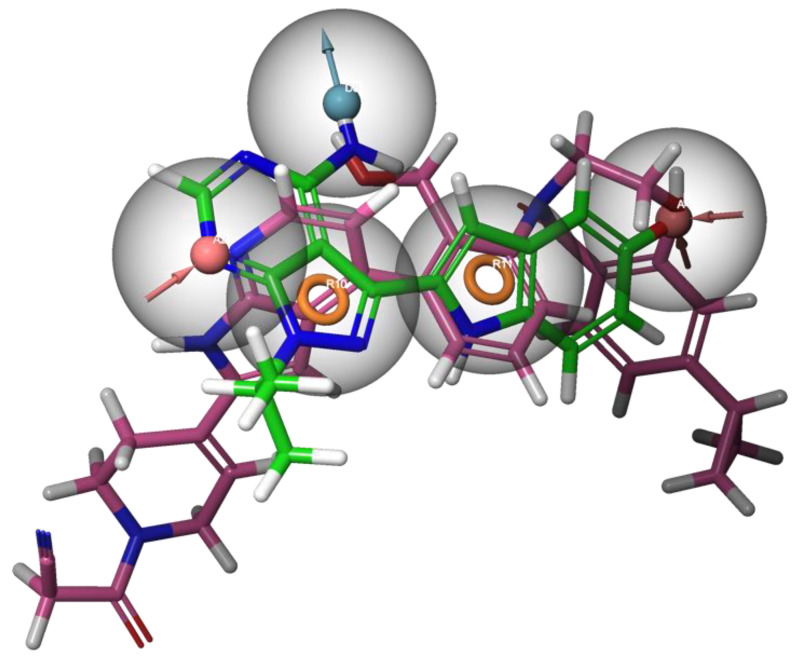
A sample pharmacophore of two known P2X7 antagonists coloured in fuchsia and green. The ligands have been clustered such that similar molecular features are aligned with each other and the pharmacophore. This sample pharmacophore constitutes five key features. Two aromatic groups are represented by the orange rings, two hydrogen bond acceptors by pink spheres and hydrogen bond donors by light blue spheres. The grey spheres surrounding these features are known as exclusion volumes, which mimic what the protein binding pocket is expected to look like. Thus, query ligands must not enter these regions to prevent steric clashes. The collection of these features is what is expected to contribute most to drug receptor interactions.

3D-pharmacophore models are comprised of molecular features, such as the steric and electrostatic properties that allow the ligand to bind to the target receptor to produce a pharmacological response. Functional groups represented as hydrogen bond donors or acceptors, charges, hydrophobic and aromatic are identified in the pharmacophore [15]. In essence, pharmacophoric modelling uses a training set of at least two ligands to generate a 3D map of the key molecular features (Figure 2), which is then validated using a test set of other active molecules and decoys [16]. Decoys can be obtained from the Database of Useful Decoys: Enhanced (DUD-E), which is a database of biologically inactive small molecules with similar physicochemical properties [17]. This technique is particularly useful as a pre-screening tool for the filtering of ultra large chemical libraries prior to further assessment using more computationally expensive methods such as molecular docking. A similar workflow was used in pursuit of human dopamine transporter (hDAT) antagonists for new Parkinson’s disease therapies. A pharmacophore was also generated using 25 known active ligands and 50 decoys as a training set and 50 substrates and 2500 decoys for the validation set. The top scoring 1000 ligands from the docking studies of 300,000 ligands then underwent pharmacophore screening, which uncovered six promising molecules. Three leads demonstrated a statistically significant inhibition of hDAT receptor uptake in biological testing.

To develop a robust model that is representative of all known active molecules, pharmacophore generation must be an iterative process whereby models are scored and refined until the most suitable solution is ascertained. Typically, the scoring and generation of pharmacophore models can be categorized into either overlay or root mean square derivative (RMSD) scoring [18]. Overlay methods generate and score a hypothesis for a pharmacophore by matching the radii of chemical features from the alignment of molecules in 3D space [19,20]. Alternatively, RMSD uses the measured distances between a functional group on a query molecule and the pharmacophore model [21,22]. Pharmacophore modelling programs are reviewed in greater detail by Sanders et al., and Giordano et al. [18,23].

### 2.3. QSAR

The concept of the quantitative structure–activity relationship (QSAR) is underpinned by the correlation between the physicochemical properties and topological features of a molecule and the biological activity they exert on a target [24]. QSAR studies use this relationship to filter and rank libraries of molecules and predict biological activity. These predictions are made possible by the use of statistical methods to correlate molecular descriptors to biological data such as binding affinity (K_D_) or functional potency (EC_50_ or IC_50_) values. Data may be obtained in-house (for example, using proprietary data), or, more commonly, chemical databases (Figure 1) are used to access data for the training and testing of QSAR models. These predictive models are especially useful when attempting to design a drug with multiple targets, such as those in the examples presented in chapter 6. QSAR modelling is also attractive for lead optimisation through the identification of areas responsible for biological activity. A 3D-QSAR model was utilised for the lead optimisation of phosphodiesterase 4 (PDE4) inhibitors that could be used for major depressive disorder [25]. Previous leads were optimised by the addition of hydrophobic and hydrogen bonding groups that extended into other pockets of the active site. In vitro assessments indicated that the new compounds had nanomolar IC_50_ values and demonstrated anti-inflammatory properties in microglial cells. 

Ideally, the molecules used in model generation should be split into approximately 80% for training and 20% for testing in addition to an external validation set [26,27]. It is important that the molecules in the training set are as chemically dissimilar as possible [27] and the bioactivity data of these chemicals is distributed across the full range of endpoints [28] to ensure validity of predictions and minimise biases. Given these molecules cannot capture the full breadth of chemical space, it is essential to define an applicability domain (AD). An AD stipulates the area of chemical space for which the model can make predictions with good reliability [29]. This is one of the five guidelines outlined by the Organisation for Economic Co-operation and Development (OECD) recommendations for valid QSAR development [26]. 

The dimensionality of molecular descriptors in QSAR may range from zero-dimensional to six-dimensional (0D to 6D). Increasing dimensions of chemical representation will increase the level of detail about molecules in the QSAR model and, subsequently, the complexity and computing power needed. The examples in this study are limited to 3D methods, and therefore dimensions 4D–6D will not be discussed. All dimensions of QSAR are explained and reviewed in detail by Manoj et al. [30]. Table 1 outlines the details of molecular descriptors from 0D to 3D [31,32]. 

Once a dataset has been curated and descriptors generated, an algorithm must be selected to complete the regression task. Broadly speaking, the algorithms used in QSAR can be categorised into linear and non-linear methods [33]. Popular linear regression tools include partial least squares (PLSs) [34]. PLSs transform large, high dimension data such as molecular descriptors into linear solutions to make predictions. Non-linear methods include k-nearest neighbours (k-NNs) [35], support vector machines (SVMs) [36] and random forest (RF) [37]. Both k-NN and SVMs use the distances between parameters in the hyperplanes to determine solutions to the QSAR regression problems. In contrast, RF uses a collection of decision trees to build one robust predictive model. 

To ensure the QSAR model being used has robust predictive abilities, validation studies must be undertaken. This is another recommendation from the OECD for the development of a credible QSAR model [26]. It has been suggested that an external validation set approximately 15 to 20% the size of the entire dataset should be used to ascertain a model’s performance [38]. R^2^ (1–(RSS/TSS), where RSS is the residual sum of squares and TSS is the total sum of squares) and q^2^ are the most popular parameters to check the goodness of fit for QSAR models [39,40]. The q^2^ value is obtained by calculating R^2^ using leave-one-out cross validation [27]. Ideally, R^2^ should be as close to 1 as possible for goodness of fit [27]; however, other studies have suggested that q^2^ > 0.5 and R^2^ > 0.6 is sufficient [38]. It is important to note that a high q^2^ does not always guarantee good external validity [27]. Alternatively, root mean squared error (RMSE) or MAE can be used, which in some cases may be better indicators of predictive ability on experimental data [41]. 

## 3. Structure-Based Methods

Structure-based drug design (SBDD) is a branch of CADD and is utilised when the 3D-structure of a biological target is available. The insight into the composition of a ligand binding site allows for the screening of compound libraries and the specific design of molecules to fit optimally to the ligand binding site. Recent advances in crystallisation techniques and technical advances in cryogenic electron microscopy (cryo-EM) means that the pool of available 3D-structures of important drug targets is rapidly expanding. Furthermore, the completion of the Human Genome Project and use of artificial intelligence-based structure prediction tools such as AlphaFold2 [42] and RoseTTAFold [43] allow for prediction of structures of proteins from sequence alone. In addition, the increase in the speed of high-performance computing and the use of graphical procession units (GPUs) means that the screening of ultra-large libraries of commercially available and make-on-demand molecules is now possible [44]. These developments in SBDD permit an expansion in the areas of chemical space that can be explored in the pursuit of new drugs and may benefit the development of drugs for the treatment of psychological and neurologic conditions. 

### 3.1. Homology Modelling and Molecular Docking

The first step in structure-based drug design (SBDD) is selecting a target structure and identifying the binding site of interest. The target structure can be determined experimentally by nuclear magnetic resonance (NMR), X-ray crystallography or cryo-EM. The Protein Data Bank (PDB) [45] is a repository for such experimentally determined structures. Alternatively, where the structures of the target of interest are not available, they can be modelled computationally using the structure of one or more evolutionary related proteins [46,47]. A minimum similarity of 30% between the template and target sequences is usually recommended [48]. The PDB contains nearly 200,000 experimentally determined structures at the time of writing, all of which can be used as templates [45]. The sequences of both the desired target and the template can be accessed via the Uniprot [49] and Swissprot [50] databases and aligned using programs such as T-Coffee [51] or BLAST [52]. Tools such as MODELLER [53] and SWISS-MODEL [54] or a commercial software such as Prime [55] and MOE [56] are used to perform the homology modelling process. In essence, the template structure is used as a basis to guide predictions of protein folding by using regions of similarity. Further details about the processes involved in homology modelling have been reviewed recently [57,58]. Typically, many models based on different starting seeds are generated and the most accurate model suitable for further studies is ascertained using scoring functions such as the discrete optimized protein energy (DOPE) [59] or qualitative model energy analysis (QMEAN) [60]. Both scoring functions are statistical analyses that assess the local and global energy potentials of a homology model to discern if the structure is realistic. Homology models of the human dopamine transporter (hDAT) [61] were an essential first step in ascertaining new leads towards treatments for Parkinson’s disease. Similarly, homology modelling assisted in uncovering dual-target ligands for the α7 nicotinic acetylcholine receptor (nAChR) and acetylcholinesterase (AChE) in the search of improved therapies for Alzheimer’s disease [62].

Molecular docking is a SBDD technique that predicts the preferred orientation and conformation of a ligand in a binding site. It can be used to screen databases of ligands, provide scores for the predicted binding energy and rank the ligands to assist in finding potential hit molecules [63,64]. In molecular docking studies, possible poses of the ligand are explored by allowing the ligand to be flexible when binding into the protein, which ensures the lowest possible energy state of each ligand–protein complex to be determined when scoring and ranking the ligands [65]. Conformers are generated either a-priori, by systematically exploring all possible degrees of freedom with respect to ligand binding, or by randomly modifying parameters, such as torsional angles, depending on the search method chosen [63]. Once ligand conformations are generated, they are then docked into a rigid protein binding site for evaluation and the scoring of binding interactions. Alternatively, induced fit docking (IFD) protocols can be used, whereby the protein exhibits a degree of flexibility. Whilst this can lead to more accurate predictions in terms of binding potential, it is significantly more computationally expensive compared to standard docking protocols [66]. Thus, it is typically used in post processing to ascertain a hit compound’s binding mode. To assess the binding interactions of ligands with dual activity against tumour necrosis factor receptor 1 (TNFR1) and inhibitor of nuclear factor kappa-β kinase subunit β (IKKB) complex, an induced fit docking protocol was employed [67]. This identified avanafil as the most promising lead compound for biological evaluation. Avanafil demonstrated neuroprotective effects in a mouse model of neuroinflammation as well as a reduction in the formation of amyloid-β plaques and inflammatory cytokines in mouse brains.

The scoring of ligands determines which ligand pose is the most energetically favourable and ranks the library of screened ligands to indicate which compounds are most likely to be active and suitable for further research [68]. This is particularly important when large chemical libraries are being screened. One study, which was investigating new inhibitors against the voltage gated sodium channel Na_v_1.7 towards new therapies for neuropathic pain, used docking studies in BioSolveIT to refine a chemical library of 1.5 million ligands down to nine leads with demonstrated efficacy in a mouse model of neuropathic pain [69]. An important secondary finding from the docking studies was that a new binding mechanism to previously described sulfonamide Na_v_1.7 inhibitors at the active site was noted with the new ligand. Generally, scoring functions can be divided into three different categories: (1) empirical [70,71,72], (2) knowledge-based [73,74] and (3) force field-based scoring functions [75]. The scores are generated either through accounting for the individual contributions of energy terms, including hydrogen bonds, electrostatics and hydrophobicity, or they are derived from statistical analysis of experimentally determined ligand–protein complexes. Regardless of the scoring function, the primary goal is to filter through large chemical libraries to find the ligands with the best properties for further research.

### 3.2. Molecular Dynamics Studies

Docking examines a single frame in time whereas, in reality, proteins and ligands are flexible over time and present in a dynamic, complex cellular environment in contact with water, membranes, and ions. Molecular dynamics (MD) can account for this as a technique either before or after docking to study protein conformation, flexibility, stability and ligand sampling [76]. Due to the ability to account for and simulate motion, MD intrinsically simulates the dynamic protein system and therefore accounts for protein structural changes, which is not possible in conventional docking studies, which are typically limited to rigid protein structures or structures with localised movements [77,78]. The validation of docking results was carried out during investigations for antagonists of LRRK2 for new treatments of Parkinson’s disease [79]. The MD simulations provided insight on the likely binding mode of a potent new ligand, LY2019-005. 

MD aims to simulate the time-based change in atom positions, using Newton’s equation of motion (F = ma) to offer a relationship between the force and acceleration of the atom dependent on atomic mass over time. These algorithms are implemented as force fields, with common examples including CHARMM [80], GROMOS [81], AMBER [82] and OPLS [83]. These force fields are implemented in molecular dynamics engines such as GROMACS [84], AMBER [85], OpenMM [86] and Desmond [87], which can support one or more of the aforementioned force fields. Recent developments of large-scale simulations using coarse-grained (CG) force fields such as MARTINI [88] are gaining traction for large membrane and organelle level studies. Furthermore, recent advances in GPU hardware capabilities and algorithms such as CUDA [89] has resulted in computing performance increases of up to 100 times faster as compared to when calculations are performed on a traditional central processing unit (CPU)-only workflow, enabling microsecond time-scale simulations. However, often the binding of ligands involves energy barriers which need to be overcome for ligand binding or conformational changes. Several methods have been devised to overcome this issue, including metadynamics, replica exchange and alchemical techniques including free energy perturbation (FEP). Details of these methods are beyond the scope of this review, but they are nonetheless critical for applications in drug design and discovery.

MD simulations require a starting point, typically experimental models based on X-ray crystallography, cryo-EM or NMR spectroscopy. Computational models such as homology modelling, described in Section 3.1, and deep learning methods such as AlphaFold2 [42] may also be utilised as a starting model where the experimental structure is not available. Where computational methods have been implemented, MD simulations may help to ensure the validity of these models. Park et al. [90] employed MD simulations to investigate the binding properties of an optimised lead compound with predicted antagonistic effects against the G2019S mutant leucine-rich repeat kinase 2 (LRRK2) receptor. The mutated LRRK2 receptor is known to contribute to Parkinson’s disease pathophysiology through increased activity. The MD simulations indicated that the protein–antagonist complex remained stable, and in vitro testing confirmed that this optimised lead had nanomolar potency.

In MD simulation preparation, membrane proteins are placed within a membrane model consisting of one or more lipid species such as phosphatidylcholine (POPC), phosphatidylethanolamine (POPE) and cholesterol. In an explicit water simulation system, individual water molecules are placed to fill the simulation box and random water molecules are removed and replaced with ions such as Na^+^ or Cl^−^. Simulation systems require several steps of processing before they can be simulated for production runs, with this including minimisation and equilibration, where the MD system is simulated for a short period to reduce the overall system energy and to stabilise the system for production simulations. Post-simulation analysis metrics include measures such as the root mean square deviation (RMSD), a measure to determine the overall structural deviation from the initial starting pose, and root mean square fluctuations (RMSF), a measure to determine the movement of individual residues in the protein to determine the flexibility of the protein and the ligand of interest. Further analysis including ligand-to-residue contact analysis, ligand environment analysis and binding pose clustering is also performed to determine potential ligand binding poses and intermediate binding sites.

## 4. ADMET Property Prediction

A key challenge in all drug discovery endeavours is ensuring that new leads have acceptable pharmacokinetic parameters of absorption, distribution, metabolism and excretion whilst also minimising toxic effects (ADMET). There exists further difficulty for CNS pharmaceuticals that must cross the blood–brain barrier (BBB), as this is a complex process that involves both the passive and active diffusion mechanisms [91]. The incorrect prediction of ADMET properties may be an extremely costly error if not determined early in the drug discovery pipeline, as unacceptable pharmacokinetic parameters have been reported to account for 40% of failures in phase II clinical trials [92]. Thus, several methodologies to predict favourable ADMET properties as part of CADD workflows exist. Much like QSAR studies, the prediction of pharmacokinetic parameters utilises machine learning techniques including SVM, k-NN and RF to ascertain the relationship between molecular descriptors and ADMET properties. The relationship between molecular descriptors and in vitro data points is used to predict the pharmacokinetic parameters that new leads would exhibit. The applications of ADMET prediction in computational drug discovery is extensively reviewed in [93,94]. Given that the focus of this review lies in the domain of neurological and psychiatric drug discovery, the remainder of this chapter will discuss advances in the computational prediction of BBB permeability.

The prediction of BBB permeability by passive diffusion can largely be predicted from physicochemical properties including lipophilicity, polarity and ionisation at physiological pH. However, several mechanisms of active transport into and efflux out of the CNS must also be accounted for in these predictions [95]. Additional data such as in vivo BBB permeability has proven useful for these considerations [96]. The use of datasets that encompass drug phenotypes including the clinical indication and CNS-related side effects, in addition to physicochemical properties, to determine BBB permeability is a similar approach [97]. This work was expanded upon by Miao et al., with a deep learning algorithm which demonstrated a marked improvement of 97% accuracy, compared to the 86% accuracy of the initial SVM model [98]. The increased accuracy can be attributed to the enhanced capacity of deep learning models to understand the abstract relationships between parameters. Work has also been carried out in the development of QSAR models that identify substrates of efflux proteins implicated in poor CNS uptake, such as the multi-drug resistance protein 1 (MRP-1) [99] and breast cancer resistance protein (BCRP) [100]. More recent applications in this area make use of image recognition [101] and natural language processing [102] advancements in deep learning to aid in BBB permeability predictions, which have resulted in accuracies as high as 99%. The underlying principles of these applications are discussed below.

## 5. The Rise of Deep Learning in Computer-Aided Drug Discovery

**Figure 3:**
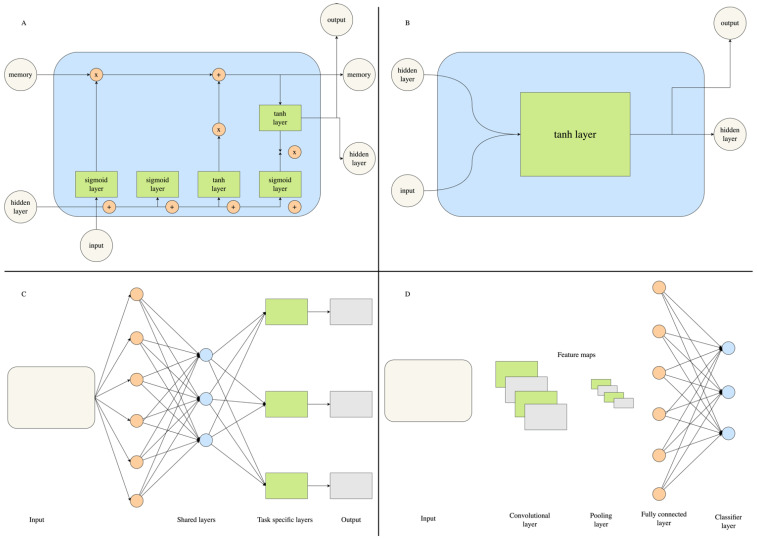
Workflows of LSTM (**a**), RNNs (**b**), multi-task learning (**c**) and CNNs (**d**).

While deep learning (DL) is not a new methodology, with it having been applied to language and image processing for several decades now, its applications to drug discovery efforts have only emerged in the last few years [103]. This has been accelerated by the use of GPUs to handle the computationally expensive calculations associated with deep learning [104]. DL extends beyond traditional machine learning methods by using several processing layers, known as neurons, to make predictions based on large collections of multi-dimensional data [105]. Biological data for different CNS targets to train DL models may be obtained from open-source databases such as ChEMBL [106], PubChem [107] and MolData [108]. In-house datasets from experimental studies are also commonplace in the pharmaceutical industry [109]. Whilst several types of deep learning architectures exist, the most prominent applications in drug design and discovery are convolutional neural networks (CNNs), recurrent neural networks (RNNs), long-short term memory (LSTM) and multi-task learning (MTL) (Figure 3). Thus, these will be the primary focus of the review. Detailed reviews on all types of deep learning and their underlying principles are provided by Le Cun et al. [105] and Schmidhuber et al. [110].

The most widely used application of CNNs are in image recognition, as the architecture mimics that of the visual cortex [111]. CNNs collect information about the presence or absence of features in different locations of images using a feature map, which is derived from convolutional layers. It is these feature maps that give CNNs the advantage in terms of spatial awareness over other DL architectures. Next, a rectified linear activation function (ReLU) is applied, which serves to account for interactions between variables and non-linearities in the model. A pooling layer will summarise the information collected in the aforementioned layers to prevent model overfitting. These steps will be repeated several times, with at least 1 fully connected layer to link the hidden layers to the output [112]. In drug design, CNNs are used to extract features from 2D or 3D molecular graphs, with them demonstrating superior performance over other machine learning and deep neural network (DNNs) methods which use molecular fingerprints [113]. The features extracted from the molecular graphs can be used to predict pharmacokinetic properties [113] or to ascertain the correct binding poses and binding affinities of ligand–protein complexes [114,115]. Graphical CNNs were employed, with images of molecule fingerprints as the input, in the search for new AChE inhibitors for the treatment of Alzheimer’s disease. The deep learning model outperformed three other machine learning methods: linear regression, random forest and XGBoost. Two hit molecules were identified from a library of 2 million small molecules. The resulting leads are able to traverse the BBB and outperformed galantamine in vitro, thus demonstrating promise as potential drugs [116]. 

Recurrent neural networks can process sequential data by linking information from one time point back to an earlier time point. This property means they require a memory buffer to store information from previous states [117]. These properties mean RNNs are excellent tools in language processing. These neural networks are cyclic in nature, meaning hidden neurons receive feedback from both the input and the memory buffer. Information about correct and incorrect outputs are fed back to the respective neurons which contributed to that prediction using backpropagation. However, RNN models are limited in that it is difficult to learn and store over very long time points given the sequential nature of the models. Long-short-term-memory is an extension of RNNs which can overcome this limitation [118]. LSTM works using a similar principle to RNNs but has a memory cell which accumulates input information by connecting to itself at future time points. In addition, the LSTM architecture contains leaky gated neurons, which learn to decide whether to clear the stored information at later time points. The most significant contribution of RNNs and LSTM to medicinal chemistry are their abilities to learn and correctly predict SMILES strings or other linear chemical notation systems. This can be used in retrosynthesis to design new drugs with feasible synthesis routes [119,120]. In addition, RNNs have been trained to interpret protein sequence data and used concurrently with graphical CNNs to predict ligand–protein affinity in the DeepAffinity model [121]. Multi-task learning uses a collection of learning algorithms and analysis methods to make predictions for multiple tasks. These tasks are learnt in parallel whilst making what is learned by each task available to the overall model through backpropagation [122]. This is particularly useful in models where the biological data being used in the models have different values from several experiments for the same target. In addition, multi-task learning has demonstrated capabilities in the prediction of drug activity against proteins from the same class (GPCR and ion channels) [123,124,125].

In computational drug discovery, as demonstrated above, deep learning applications are producing advances over more traditional techniques. Novel algorithms which combine both CNNs and LSTM for de novo design, such as the RELATION model [126], show promise in finding novel leads from an expansive chemical space. A similar application exists specifically for CNS drug design [127] which also accounts for the added complexity of BBB permeability. The benefit of applying DL techniques to computational drug discovery is the ability to better process the complexity of molecular descriptors and their interactions with biological systems. Neural networks and other machine learning-based methods have also been utilised to understand the relationships between genes, an individual’s environment and disease biomarkers for mental illnesses such as schizophrenia and depression as well as Alzheimer’s disease [128,129,130,131]. These modelling techniques are aimed towards precision medicine and enhanced disease understanding for improved therapeutics. In addition, DL architectures require large quantities of data for high performance and therefore are well positioned to handle information from large databases. Although DL applications in neurological and psychiatric drug discovery are still emerging, there is clear potential for this application to enrich drug discovery efforts. 

## 6. Applications to Neurological and Psychiatric Conditions

Computational methodologies have become an essential part of drug design. Recent examples have demonstrated the potential of these techniques in terms of accelerating the drug discovery pipeline and reducing the time and money spent on laboratory testing and clinical trials. Table 2 provides an insight into how CADD techniques are being applied to develop drugs targeting neurological and psychiatric conditions, and other examples are given in the text below. From our search of the literature, Alzheimer’s disease was the most prominent disease target, with several CADD studies using a diverse range of drug targets being reported below. Given that over 55 million people globally suffer from Alzheimer’s disease, with the World Health Organisation projecting this to more than double over the next 30 years [132], the need for new treatments is imperative. The applications of CADD studies for Alzheimer’s disease exemplifies this demand. Schizophrenia was the most common psychiatric disease target in the review of the literature, with targets such as α7 nAChR selected to improve available treatment options for sufferers. It is important to note that even though a number of the distinct therapeutic areas discussed below share similar target proteins, these possess vastly different clinical phenotypes. The relationship between shared disease genotypes with distinct clinical manifestations can be accounted for, in part, by epigenetic factors [133] and is beyond the scope of this paper. This section will discuss the therapeutic areas where CADD studies for CNS diseases have been reported, with Table 2 providing further exemplars (presented below).

### 6.1. Alzheimer’s Disease

**Figure 4:**
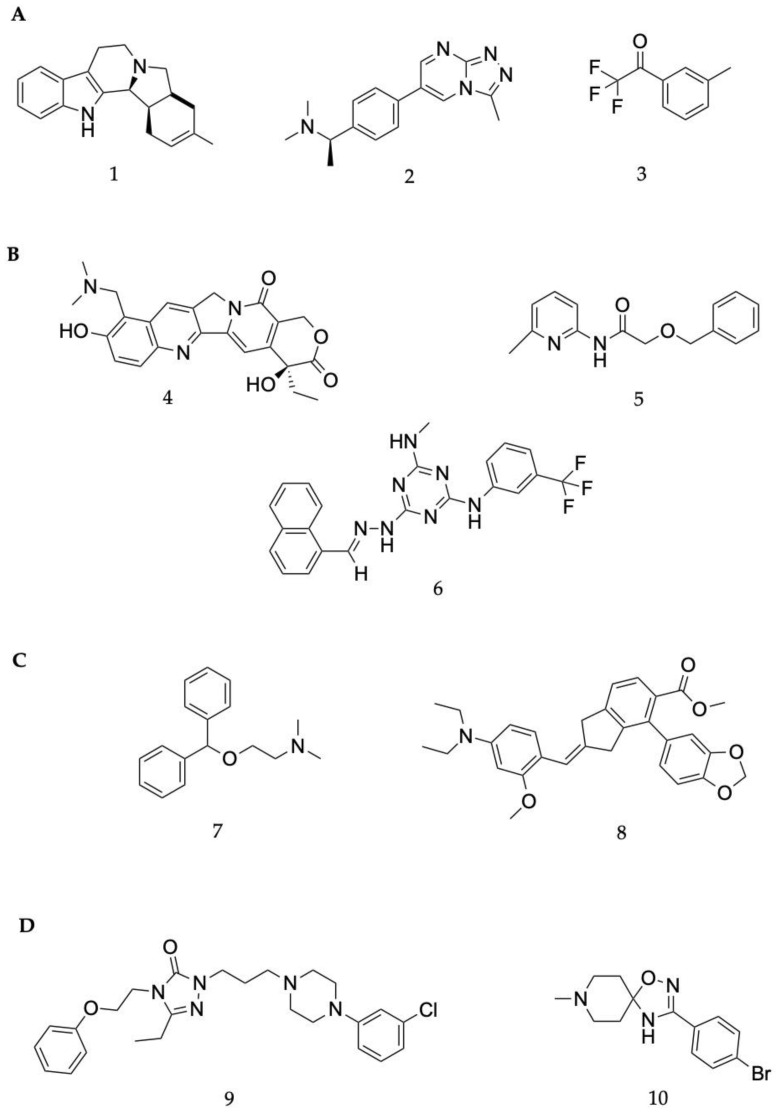
Chemical structures of lead compounds with the highest reported in vitro activity from exemplar papers discussed below. The molecules are proposed to be of use in therapeutics for Alzheimer’s disease (**A**), Parkinson’s disease (**B**), neuropathic pain (**C**) and schizophrenia (**D**).

A multi-target activity 3D-QSAR model against acetylcholinesterase (AChE), serotonin transporter (SERT), beta-secretase 1 (BACE1) and glycogen synthase kinase-3 (GSK3β) was built to towards new therapeutics for Alzheimer’s disease [134]. The QSAR model was built using IC_50_ data from ChEMBL [106], with both a multilinear regression and an artificial neural network (ANN) model being used. The 2D structures of ligands were converted to 3D using OpenBabel, and molecular descriptors were generated using FQSARModel. During model validation, it was apparent that ANN models performed better, and these were therefore selected to be used in virtual screening. Over 20,000 compounds from the ZINC (biogenic) database were docked against all four proteins. After docking in both Glide and Autodock, 57 compounds with drug properties and favourable ligand efficacy were then screened against the QSAR models. The models indicated that five ligands held promise in terms of targeting at least three of the four proteins. One compound, ZINC4027357 (**1**, Figure 4A), demonstrated the inhibition of both AChE and BACE1. None of the selected hits had inhibitory properties against SERT or GSK3β within the selected potencies.

A structure-based application of the multi-target approach was also used to identify lead compounds with dual activity against AChE and α7 nAChR. The ZINC15 database [135], consisting of over 7.5 million small molecules, was filtered to remove molecules with unfavourable properties such as Lipinski’s violations, resulting in 3.8 million ligands being selected for virtual screening in Glide targeting the human AChE and an α7 nAChR homology model, which was built using MODELLER. There were 57 compounds shared between both proteins, of which 16 were selected for in vitro assessment. Compound Ymir-2 (**2**, Figure 4A) possessed the most favourable chemical profile and dual target activity [136].

A deep learning approach based on a series of regression models that were built with the aim of predicting binding free energy towards AChE was produced. Of the regression models, a graphical CNN model had the best results, with an RMSE of (1.580 ± 0.137 kcal mol^−1^). This model was selected to screen a dataset of 2 million compounds, of which 6 were identified as suitable for docking with AutoDock Vina, MD simulations using GROMACS and in vitro assessment. Benzyl trifluoromethyl ketone (**3**, Figure 4A) outperformed galantamine, with an IC_50_ value of 0.33 μM against AChE. Permeability assessments suggested these ligands may traverse the blood–brain barrier [116].

### 6.2. Parkinson’s Disease

A drug repurposing study aimed to find new Parkinson’s disease treatments using associations between approved drugs and proteins associated with Parkinson’s disease. The CNN model demonstrated superiority against other benchmark approaches (e.g., DTINet and deepDTnet), with an accuracy of 91.57%. In addition, the CNN model outperformed traditional machine learning algorithms. The top 10 ranked compounds from the unknown samples underwent molecular docking against the 5-hydroxytryptamine receptor 2A (5HTR2A) to ascertain favourable interactions between these ligands and the target proteins. Pimvanserin was used as a positive control, for which three of the ten ligands had comparable binding energy, of which the topoisomerase inhibitor topotecan (**4**, Figure 4B) was the most promising [137].

Another deep learning approach, using deep neural network architecture was built to identify piperine-like compounds and drugs against these targets. The model demonstrated an accuracy 87.5%. A total of 57,423 compounds from the ZINC and PubChem databases underwent a similarity search based on piperine to find similar structures. In all, 101 compounds were selected for further investigation through docking in AutoDock 4.0, of which 5 were suitable for MD simulations on the AMBER platofrm. The docking and MD studies revealed that an additional ring in top performing compounds (**5**, Figure 4B) is likely to help to form hydrogen bonds in the active site, which leads to a greater potency against Monoamine-oxidase A and B (MAO-A and MAO-B) [138].

A docking study of over 1.6 million small molecules was conducted against a homology model of LRRK2, which was conducted using both the Glide and Prime modules of Schrodinger’s Maestro software. In total, 28 high performing molecules were purchased for biological evaluation. Two small molecules with novel features, namely LY2019-005 and LY2019-006, were identified, with these also being able to pass the blood–brain barrier. MD simulations were conducted to investigate the binding mode of these ligands. Both ligands possessed nanomolar IC_50_ values for both the wild-type and G2019S mutant enzyme, with LY2019-005 (**6**, Figure 4B) being the most potent. Given the neurotoxic potential of the G2019S mutant of LRRK2, nanomolar IC_50_ is particularly important [79].

### 6.3. Neuropathic Pain

The biological data of 180 sigma-1 receptor (S1R) antagonists was curated from the literature and split into training and test sets in a 4:1 ratio, with the randomization of this process repeated 50 times to avoid bias. MOE software was used to generate 206 molecular descriptors, which then underwent dimensionality reduction using principal component analysis (PCA). An atom-based 3D-QSAR model was developed using the partial least squares technique, with the final model having an RMSE of 0.29 and R^2^ of 0.92, both of which are suggestive of a model with good predictive abilities. An energy-based pharmacophore was also developed to supplement the 3D-QSAR model by providing conformational information about the binding site. This was used in the virtual screening studies to ensure the ligands were in the correct orientation for the binding site, developed using Schrödinger’s Glide. After pre-filtering steps, 1935 FDA-approved drugs from the DrugBank database were initially screened against the pharmacophore model, with the best fitting conformer of each ligand then being screened in the 3D-QSAR model. Twelve of the best performing ligands with no prior biological affinity data against S1R underwent further assessment in vitro using a radio–ligand binding assay. Two drugs, phenyltoloxamine and diphenhydramine, exhibited 66 and 70% inhibition at a concentration of 1 μM, respectively. This also conferred with reports in the literature that diphenhydramine (**7**, Figure 4C) can be used as an adjective analgesic [139].

A highlight on target sequence (HoTS) deep learning model was used to scan the purinergic P2X3 protein sequence to identify new binding sites in search of treatments for neuropathic pain [140]. Once the DL model had identified potential binding sites, MD simulations in the CDOCKER software package were used to measure the volume of the binding sites to ensure their feasibility. Four new binding regions were identified, for which a pharmacophore model was developed using the binding mode of a known antagonist and its derivatives in the BIOVIA software. Over 97,000 compounds were screened against the pharmacophore model. A total of 2346 ligands were then docked to assist in prioritisation for in vitro assessment, of which 500 were selected for experimental validation. A total of 16 compounds with novel structures and low micromolar IC_50_ values were identified. Compound **8** (Figure 4C) was the most potent lead compound.

### 6.4. Schizophrenia

A 2D-QSAR model was developed using 159 inhibitors of the sigma 2 receptor (S2R) reported in the literature. MOE software was used to generate molecular descriptors of each ligand for QSAR model generation. Four algorithms were generated, namely stepwise regression, Lasso, genetic algorithm (GA) and an algorithm, GreedGene, which was developed by the authors. GreedGene had the best performance, with an R^2^ of 0.56, and was selected for screening. A pharmacophore model was also generated using Glide for use in virtual screening. Over 2000 small molecules from the DrugBank database were screened against the QSAR model, which had a pKi cut-off of 5.5. A total of 823 ligands were then screened against the pharmacophore model, before the best 120 underwent shape-based screening. Ligands that shared similarities to siramesine and ligands with a piperazine-containing scaffold or tetrahydroisoquinolinyl structures were kept for in vitro testing. These scaffolds are known for high S2R binding affinity. A total of 30 compounds possessed this scaffold and were identified as promising leads. Six molecules underwent biological testing, which revealed three FDA approved drugs, nefazodone, cinacalcet and pimozide, had nanomolar binding affinity values, with nefazodone (**9**, Figure 4D) being the most potent of the three [141].

A pharmacophore was generated using 11 α7 nAChR agonists from the literature. The pharmacophore consisted of a hydrogen bonding region, a hydrophobic centre and one positively ionised group. To reduce the number of false positives, a recursive partitioning model was also used. A virtual screening of the ChemDiv database against these two models was performed. After filtering to ensure no Lipinski parameter violations, 13 ligands were selected for in vitro assessment, 10 of which had demonstrated inhibitory effects. T761-0184 was selected for further investigation due to its high potency. This ligand underwent induced fit docking to a homology model of α7 nAChR to ascertain the binding mode for structural optimisations. Of the 51 optimised structures, B10 (10, Figure 4D) exhibited subtype selectivity for α7 nAChR over other nAChR subtypes. B10 was also one of the most promising ligands, with an IC_50_ value of 5.4 µM [14].

**Table 2:** Further examples of computer-aided drug discovery for new neurological and psychiatric treatments from the literature.

Drug Target and Methodology	Study Significance	Chemical Structure	Reference
**Drug target:** Transient receptor potential sub family M4 receptor (TRPM4) **Disease target:** Multiple sclerosis **Software packages:** CORINA **Methods:** xLOS	A ligand-based screening method known as atom category extended ligand overlap score (xLOS) was used to ascertain leads from a library of over 900,000 small molecules. This method was chosen due to a lack of information about the structure and binding pocket of TRPM4. Three reference compounds and the database compounds were converted into 3D structures using CORINA software. xLOS was then used to compare database ligands to the reference compounds, 9-phenanthrol, glibenclamide and flufenamic acid, and rank them. A total of 214 of the top molecules were purchased for biological evaluation. An additional round of xLOS screening on the Princeton database was performed using the top three hits from the first round of biological evaluation. The biological evaluation was conducted on 247 ligands from the second round of screening. The top scoring lead had potency at approximately 1 μM IC_50_, which is a marked improvement over the initial reference compounds.	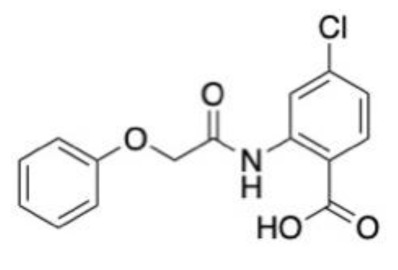	[142]
**Drug target:** N-methyl-D-aspartate receptor (NDMA) GluN1-GluN2A subunits **Disease target:** Epilepsy **Software packages:** Molinspiration Cheminformatics AutoDock 4 **Methods:** Docking	In silico ADMET assessments and docking studies revealed three compounds with acceptable pharmacological properties, including the ability to traverse the BBB. These compounds demonstrated similar binding interactions to endogenous ligands but with improved binding capacity. The lead compounds resulted in a reduced number of seizures observed in a mouse model of epilepsy without any adverse effects on motor activity.	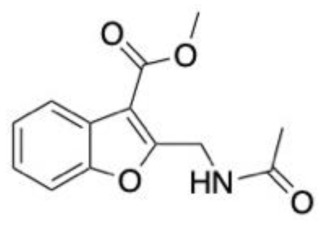	[143]
**Drug target:** Cannabinoid receptor 1 (CB1) **Disease target:** Substance abuse disorders **Software packages:** Glide **Methods:** Docking	A VS study was performed against the CB1 receptor using a natural products subset of the ZINC12 database. Nearly 300,000 small molecules were filtered and docked. The filtering and docking using standard and extra precision settings in Glide indicated 32 top-performing ligands, of which 18 were selected for further in vitro testing through clustering to ensure structural diversity amongst hits. Of the 18 ligands, 7 demonstrated more than 50% displacement in competitive binding at 10uM. Compound 16 had the greatest potency as a selective inverse agonist. Ligands with 80% similarity to compound 16 were screened and assessed for CB1 and CB2 activity. Two ligands were identified that had nanomolar affinity towards CB1. This provided key information for further structural optimization for inverse agonists targeting CB1.	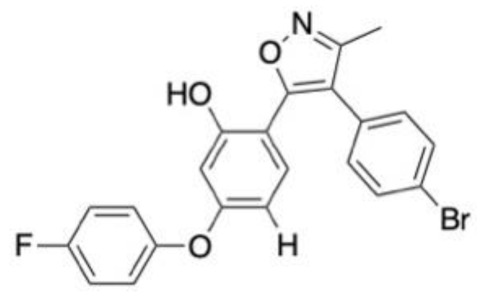	[144]
**Drug target:** Caspase-1 **Disease target:** Febrile seizures **Software packages:** Glide AMBER 14 **Methods:** Docking Molecular dynamics	The role of caspase-1 in febrile seizures was initially assessed. Mice with the caspase-1 gene knocked out did not develop febrile seizures, and their wild-type litter mates had an increase in caspase-1 prior to the onset of a febrile seizure. One million compounds from the ChemBridge database were docked against the active site of capase-1. The top 2000 ligands from the extra precision docking stage were filtered to ensure they had suitable drug properties. The remainder were clustered for chemical similarity using the Tanimoto co-efficient. Fifty ligands were purchased for experimental validation of predicted binding affinity. Four compounds had potent inhibitory effects on caspase-1. When compared to diazepam, the top compound, CZL80, showed a capacity to prevent the onset of a second episode of FS, with diazepam not being able to do this. CZL80 also reduced the risk of adult epilepsy when administered after an episode of febrile seizures.	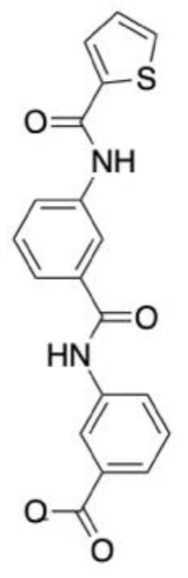	[145]
**Drug target:** Phosphoglycerate kinase-1 (PGK1) **Disease target:** Stroke **Software packages:** Discovery Studio LibDock Glide Canvas **Methods:** Docking	More than 73,000 small molecules from the Specs natural compounds and PubChem databases were docked against PGK-1 in search of agonists to protect against brain damage in stroke patients. The initial library was filtered to confirm that the small molecules possessed drug-like properties. The remaining 35,414 ligands underwent HTVS in LibDock and the remaining top 4% were docked using extra precision (XP) in Glide. The highest ranked 20% of ligands from XP docking were clustered to ascertain chemical similarity amongst hits. A total of 19 compounds from the different clusteres were selected for experimental validation. Two ligands, 7979989 and Z112553128, were noted as potential PGK1 activators as demonstrated in a *Drosophilia* oxidative stress model.	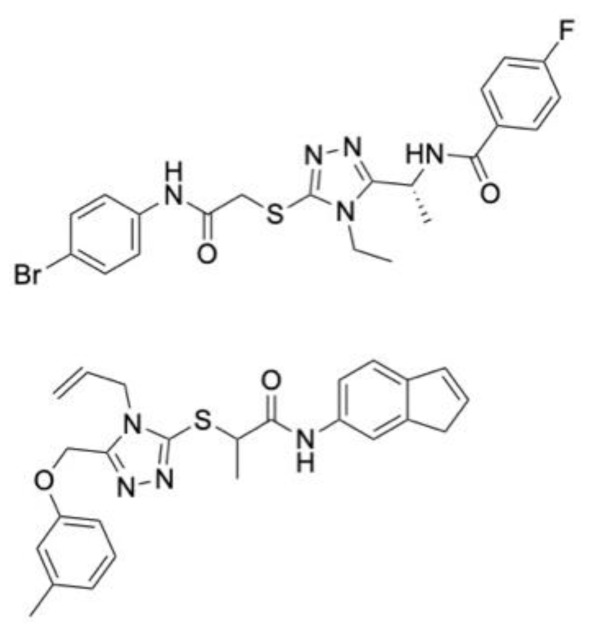	[146]
**Drug target:** Metabotropic glutamate receptor 5 (mGlu5) **Disease target:** Fragile X syndrome Depression **Software packages:** DOCK3.6 **Methods:** Docking	A total of 6.2 million compounds and fragments from ZINC database were screened to search for negative allosteric modulators (NAMs) of mGlu5. Initially, docking was benchmarked using an initial library of known NAMs and decoys with structural similarities. From this, 59 leads and 59 fragments were identified for experimental validation. In vitro assessments identified 11 identified molecules as NAMs. Compound F1 demonstrated the greatest level in terms of novelty in a pairwise Tanimoto co-efficient assessment with other mGlu5 ligands on the ChEMBL database. F1 also had the greatest affinity, with an K_i_ of 0.43μM.	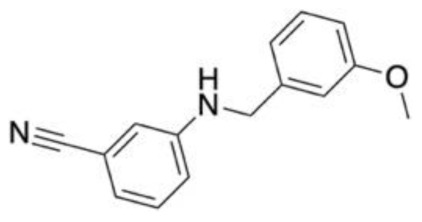	[147]
**Drug target:** Excitatory amino acid transporter 2 (EAAT2) **Disease target:** Stroke Brain trauma Neurodegenerative disorders **Software packages:** MODELLER Desmond Sybyl 8.1 Unity GOLD **Methods:** Homology modelling Molecular dynamics Hybrid structure-based pharmacophore Docking	MD studies performed on a homology model of the EAAT2 suggested the presence of an allosteric binding site. Five key residues from the allosteric site were identified as key binding residues through site-directed and functional mutagenesis studies. The virtual screening of 3 million small molecules was performed against this pharmacophore. After virtual screening and filtering for favourable ADMET properties and no Lipinski’s violations, 58 ligands were selected for docking against the EAAT2 homology model. The docking studies yielded 10 molecules of interest for further assessment. A SciFinder search confirmed the novelty of these ligands. In vitro testing confirmed four compounds as NAMs, three as PAMs and three as inactive against EAAT2. One of the top performing molecules, GT949, possessed nanomolar potency.	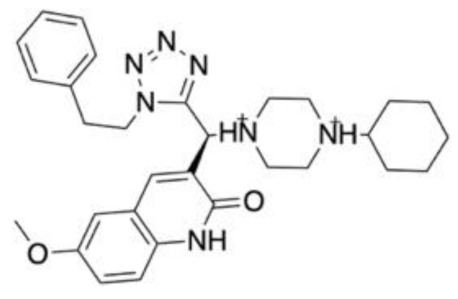	[148]

## 7. Conclusions

Computational drug design is a powerful tool in the pursuit to discover new therapies for neurological and psychiatric conditions. This literature review presents a distillation of the computational approaches to drug design from multi-disciplinary research articles to highlight the importance of CADD in finding new CNS drugs. The exemplars provided demonstrate how CADD methodologies have been utilised to present a basis for future drug development. It is apparent from the literature presented that progress towards novel therapies in CNS drug discovery is being made, in particular for drug targets where there are no therapeutics available. It is hoped that by creating novel medications towards these new targets, the standard of patient care will improve by both increased treatment efficacy and reducing side effects. In the context of CNS computational drug discovery, there is a clear trend that the majority of research is focused on investigating new treatments for neurodegenerative disorders such as Alzheimer’s and Parkinson’s disease. However, psychiatric conditions such as schizophrenia and substance abuse disorder, as well as neurological conditions such as brain injuries and neuropathic pain, are other research foci, all of which present significant disease burdens for both the patient and society as a whole. Although it is evident there is still a long way to go for new lead molecules to become approved drugs, CADD is helping to hasten this process. As newer techniques such as deep learning become more mainstream within academic drug design research, it is expected that the efficiency with which studies can be carried out will greatly increase.

## Figures and Tables

**Table 1:** Methods for the generation of molecular descriptors for QSAR modelling.

Dimension	Definition
0D	Only contains the molecular formula. Thus, the only information is the atom types and numbers of each.
1D	Molecular properties that pertain to the entire chemical structure, such as logP and pKa. It also includes substructural details of molecular fragments.
2D	Topologies are mathematically encoded to represent the connectivity of atoms using a 2D graph.
3D	Details of the spatial arrangement of atoms and non-covalent interaction sites guided by 3D topologies.

## Data Availability

Not applicable.
